# “Building the plane as you fly”: Simulation during the COVID-19 pandemic

**DOI:** 10.1017/cem.2020.398

**Published:** 2020-05-13

**Authors:** Timothy Chaplin, Tamara McColl, Andrew Petrosoniak, Andrew Koch Hall

**Affiliations:** * Department of Emergency Medicine, Queen's University, Kingston, ON; † Department of Emergency Medicine, University of Manitoba, Winnipeg, MB; ‡ Department of Medicine, University of Toronto, Toronto, ON

**Keywords:** COVID-19, education, emergency medicine, simulation

The 2019 coronavirus disease (COVID-19) pandemic is challenging our Canadian emergency departments (EDs) in unparalleled ways. As part of the frontline response, EDs have had to adapt to the unique clinical difficulties associated with the constant threat of COVID-19, developing protocols and pathways in the setting of limited and evolving information. In addition to the disruption of routine clinical care practices, an underlying perception of danger has resulted in a challenging clinical environment in which to make time-sensitive, high-stakes decisions. This has created an urgent need for targeted and adaptive training for all members of the emergency medicine healthcare team. The following commentary reflects the perspective of four emergency medicine simulation educators during the Canadian response to COVID-19. Recognizing that local needs and resources will vary, we highlight three key roles that simulation can play in the adaptive response to COVID-19: protocol development and system testing, provider education, and team-based training. The disruption to our practice as a result of COVID-19 has required us to “build the plane as we fly,” and we believe simulation to be a key tool in this process.

## SIMULATION FOR PROTECTED PROTOCOL DEVELOPMENT AND TESTING

This pandemic has brought us to uncharted waters and has required the creation of new processes and protected protocols for intubation, code blue responses, and workflows within the ED. Our familiar and reliable system 1 thinking (fast, automatic, unconscious thought processes) frequently applied during high risk events has been supplanted by system 2 thinking (slow, effortful, and deliberate thought processes) due to team safety risks related to viral exposure and infection.^1^ For example, the cognitive load required to perform common tasks, such as endotracheal intubation, has increased considerably, creating opportunities for error. In order to support system 2 thinking and mitigate error, protocols and corresponding checklists are required. Simulation allows for a safe, controlled, and iterative approach to protocol design and is well established in this context.^2,3^ It seems only natural to apply our experience using simulation for systems testing to ED preparedness for COVID-19.

Much like crash testing a car, simulation affords an opportunity for ED and simulation leaders to observe, reflect, and refine proposed protocols without risking harm to the healthcare team (or patient). This process is important to ensure a consistent approach and anticipate downstream implications prior to operationalization. Integrating the Plan-Do-Study-Act conceptual framework for systems improvement into pandemic simulation activities allows for a rapid analysis of novel protocol development. In turn, this enhanced simulation strategy serves to identify the safest and most efficient and pragmatic approach to various emergent situations. Novel pandemic-related protocols and various critical event checklists (e.g., COVID-19 intubation checklist) are now being locally trialed in a Plan-*Simulate*-Study-Act sequence prior to final implementation. Importantly, we also require the flexibility of an iterative approach as the pandemic and disease prevalence evolves, new literature emerges, guidelines are amended, and ED patient volumes change.

## SIMULATION FOR EDUCATION

The COVID-19 pandemic has created an urgency for training, fueled by both uncertainty and a risk of infection to the healthcare team. At several sites across Canada, this urgency has helped overcome typical barriers to simulation-based education, such as faculty buy-in, time, and resource allocation. As a result, EDs across the country are shifting resources, equipment, and personnel from existing programs of simulation-based education^4,5^ and implementing COVID-19-focused simulation-based training for their clinical teams at an unprecedented pace (e.g., some locations are conducting daily simulations within their departments).

In accordance with established educational paradigms, the following approaches can be used to optimize simulation-based training during the COVID-19 pandemic. Firstly, in order to maximize the educational efficiency of each session, facilitators should focus on only one or two explicit “take-away points” (e.g., minimize aerosol generation and maximize first-attempt success) and avoid long lists of objectives that won't “stick.” Furthermore, it is critical to ensure that these take-away points are clearly and immediately relevant to participants’ clinical work. Secondly, effectively learning novel COVID-19 protocols requires deliberate practice, where motivated individuals repeatedly practise, then reflect and refine their techniques.^6^ Importantly, a single simulation session will not suffice, and opportunities for practice with observation and coaching should be encouraged at the departmental level. Thirdly, different simulation modalities can be leveraged to meet a variety of training objectives. For example, individualized skill-based training in the simulation centre can be coupled with team-based interprofessional in situ simulations in the ED to maximize learning and retention. Additional layers of an educational simulation program might include resources for mental rehearsal^7^ or supporting the integration of technology through video-recorded sessions^8^ to broaden exposure and engagement.

## SIMULATION FOR TEAM-BASED TRAINING

Simulation activities should reflect the team-based reality of emergency medicine. From scenario design to delivery, interprofessional representation will result in a better “product” and outcome.^9^ This collaborative approach leverages the expertise and perspectives of all participant groups and reinforces the value of a team-based approach. For example, to reduce cognitive load and assist with clinical decisions, protected protocols and COVID-19 intubation checklists have been developed. However, if these tools are understood by only one team-member or discipline, it is unlikely that they will serve their intended purpose. In fact, they may contribute to further confusion. Interprofessional team-based training is required in order to operationalize these tools and result in a shared mental model that is critically important to high-functioning teams.^10^

COVID-19 does not discriminate, and the entire ED team assumes some degree of risk of infection as well as the associated stress. However, clinical decisions are often made by a single physician, and these decisions may influence the risk to the entire team. For example, if a physician requests bag-valve-mask ventilation, this may result in aerosol generation and potentiate the risk of infection to all team members. As a result, it is essential for everyone to understand and follow a rehearsed and standardized approach to anticipated high-risk presentations (i.e., hypoxia, cardiac arrest). If properly executed, team-based simulation not only reinforces positive behaviours, but also increases morale by bringing people together with a common goal and facilitating discussions that have benefits beyond the scenario objectives alone.

## ADDITIONAL CONSIDERATIONS

When using simulation to support ED response and preparedness to the COVID-19 pandemic, there are additional considerations that should be noted. Firstly, there is a tension between the need to practise using personal protective equipment (PPE) and the need to conserve PPE as a limited resource. We suggest that the use of full PPE be limited to scenarios in which donning/doffing is the objective and consider reusing PPE when appropriate. Secondly, physical distancing can be challenging or impossible during simulation scenarios. However, the risk of transmission can be minimized through the use of limited PPE and distancing whenever possible. Video-recording sessions, disseminating “lessons learned,” and reinforcing teaching points using other platforms can also help minimize personnel involved in each session. Thirdly, inconsistent messaging from simulation instructors can result in the spread of misinformation and distrust in the process. This can be mitigated by coordinated instructor training to ensure consistency and “scripts” to guide common questions. Further, institutional alignment with hospital leadership and infection prevention and control guidelines is important because inconsistency between departments in the same hospital can breed skepticism and concern. Lastly, it must be highlighted that there can be unintended consequences of well-intentioned, simulation-based activities. For example, the singular focus on the procedure of a protected intubation risks creating a procedural momentum that is hard to overcome. We may forget or overlook the need to discuss the indications to engage in this high-risk procedure. It is imperative, therefore, that simulations reinforce fundamentals and encourage critical thinking in high-stakes situations.

## SUMMARY

Simulation is well-suited to address many of the challenges presented by the COVID-19 pandemic to emergency medicine. As a tool for protocol development and refinement, an educational platform, and a catalyst for team-based training, simulation offers a robust and comprehensive strategy that is welcome in these uncertain times. COVID-19 has shone a spotlight on simulation as an adaptive tool, and we hope that this attention and support continue to improve patient care and build community long after the plane has landed.
